# In Situ Growth of MIL-100(Fe) on Coconut Shell Activated Carbon for High-Efficiently Removal of Microplastics from Water

**DOI:** 10.3390/polym18060772

**Published:** 2026-03-23

**Authors:** Qianyi Wang, Guohan Wang, Sasa Ma, Zichen Wang, Lijie Luo, Yongjun Chen

**Affiliations:** Department of Materials Engineering, School of Materials Science and Engineering, Hainan University, Haikou 570228, China; 23220856010003@hainanu.edu.cn (Q.W.); wangguohhh@163.com (G.W.); masasacc@163.com (S.M.); wangzc19987@163.com (Z.W.)

**Keywords:** coconut shell activated carbon, metal–organic framework, MIL-100(Fe), polystyrene, removal mechanism

## Abstract

The widespread use of plastics has inevitably led to the accumulation of persistent plastic debris in aquatic systems, where gradual fragmentation generates microplastics (MPs) that threaten ecological and biological health. Their small size, chemical stability, and resistance to degradation make effective removal particularly challenging. In this work, a composite adsorbent was fabricated through the in situ solvothermal growth of Materials of Institute Lavoisier 100 (Iron) (MIL-100(Fe)) onto coconut shell-derived activated carbon (CSAC), yielding a monolithic material denoted as CSAC@MIL-100(Fe). The integration of porous C with a metal–organic framework created a hierarchically structured adsorbent rich in accessible binding sites. The composite achieved a maximum polystyrene (PS) removal efficiency of 97.4% and maintained 91.44% efficiency after seven regeneration cycles. Stable adsorption performance was observed across a broad pH range. Structural and chemical analyses (scanning electron microscopy (SEM), Brunauer–Emmett–Teller (BET), X-ray diffraction (XRD), Fourier transform infrared spectroscopy (FTIR), X-ray photoelectron spectroscopy (XPS)) combined with adsorption modeling revealed heterogeneous multilayer adsorption behavior consistent with the Freundlich isotherm and pseudo-second-order kinetics. π–π interactions, electrostatic attraction, and coordination effects jointly governed PS capture. The Langmuir maximum adsorption capacity reached 746.27 mg/g. These findings demonstrate a practical and recyclable strategy for efficient MP remediation in aquatic environments.

## 1. Introduction

The rapid expansion of plastic production has reshaped modern society, yet its environmental legacy is increasingly difficult to ignore. Most conventional polymers persist for centuries; common plastics require approximately 100–200 years to degrade, and materials such as polyethylene terephthalate (PET) bottles may remain intact for more than 500 years in natural settings [1]. Rather than fully decomposing, discarded plastics undergo gradual weathering driven by ultraviolet (UV) radiation, thermal fluctuations, and mechanical abrasion. Over time, larger debris fragments into progressively smaller particles. Fragments measuring less than 5 mm are defined as microplastics (MPs) [2], while particles below 1 μm are categorized as nanoplastics (NPs). MPs generated through environmental breakdown are termed secondary MPs, distinguishing them from primary MPs that are intentionally manufactured at microscopic scales. Since large-scale plastic production began in the 1950s, cumulative global output has exceeded 9.5 billion tons. Recent studies indicate that for the treatment of waste plastics, incineration has gradually emerged as the dominant disposal method, accounting for 34%; the proportion of landfill disposal has decreased substantially to 40%, while the global plastic recycling rate has remained stagnant at 9% [3]. As fragmentation proceeds, these materials transform into persistent micro-sized particles. Owing to their high surface-area-to-volume ratio, MPs readily adsorb heavy metals [4] and organic contaminants [5], facilitating their transfer through aquatic food webs. MP particles have now been documented in more than 100 marine species [6,7], including organisms inhabiting the Mariana Trench. The scale and persistence of this contamination underscore the urgency of addressing MP pollution at a global level.

MPs present considerable diversity in composition, origin, and spatial distribution. Among the most frequently detected polymer types are polystyrene (PS), polyethylene (PE), polyvinyl chloride (PVC), and polypropylene (PP) [2]. In marine environments, a substantial proportion of plastic debris is associated with aquaculture and fisheries-related activities. Materials such as PE buoyancy devices, fishing nets, feed packaging, and discarded plastic bags gradually deteriorate under environmental exposure. Regional investigations along the coastline extending from Guangdong to Hainan Province, with particular emphasis on the Qiongzhou Strait, have consistently identified PS and PE as the dominant MP constituents. Field observations further reveal elevated concentrations of PS foam fragments and plastic packaging waste in nearshore waters adjacent to fishing communities in Haikou.

Current methods for MP removal from aquatic systems generally fall into three broad categories: physical separation, biological degradation, and chemical transformation. Physical approaches encompass coagulation-flocculation [2], gravitational sedimentation, membrane-based filtration, and adsorption processes [8]. Biological treatments rely primarily on enzymatic biodegradation mediated by specific microorganisms [9], whereas chemical methods most commonly involve photocatalytic oxidation. Despite their conceptual diversity, these techniques often exhibit limited effectiveness toward micro-nanoplastics (MNPs) smaller than 10 μm [10]. Sedimentation, governed by density differences and gravitational settling, achieves removal efficiencies between 40 and 90%, depending on particle characteristics [11], yet it generates secondary sludge that requires additional handling. Membrane filtration methods—including ultrafiltration, nanofiltration, and rapid sand filtration—provide effective size exclusion for MNPs below 5 μm [12]; however, membrane fouling, high operational costs, and extended processing times constrain large-scale implementation. Biodegradation offers a potentially sustainable pathway but is hindered by prolonged treatment periods (7–40 days), limited microbial adaptability, and incomplete mechanistic understanding [13]. Photocatalytic systems, although environmentally attractive, frequently result in only partial degradation under UV irradiation due to insufficient light penetration and energy transfer in aqueous environments [14]. In contrast, adsorption-based removal combines operational simplicity, relatively low cost, and facile regeneration [15], making it a promising strategy for efficient MP capture with minimal secondary environmental burden.

**Scheme 1:**
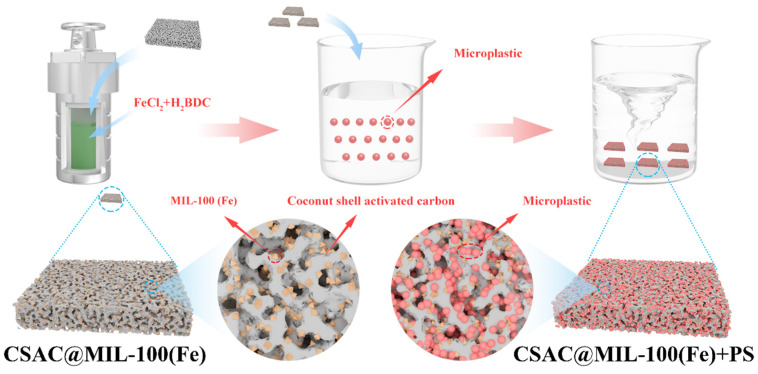
Schematic of CSAC@MIL-100(Fe) material synthesis route and PS adsorption process.

Although numerous materials have been reported for the removal of PS MPs, several practical limitations remain unresolved. Many existing adsorbents involve multistep or energy-intensive synthesis routes, exhibit moderate adsorption capacities, or suffer from poor recyclability. Moreover, investigations frequently focus on low-concentration model systems, with limited evaluation under high MP loadings or in chemically complex water matrices. In particular, the influence of coexisting ions—especially anions—on adsorption behavior has not been sufficiently clarified. Addressing these issues requires the development of materials that combine synthetic simplicity, high adsorption efficiency, structural stability, and reliable regeneration performance. PS is widely used in packaging, insulation, and consumer products, making it one of the most frequently detected MPs in aquatic environments. Its hydrophobicity and chemical stability render natural degradation extremely slow, thereby complicating remediation efforts. Despite the structural advantages of metal–organic frameworks (MOFs), the application of MIL-100(Fe) for PS removal has received limited attention. In this study, MIL-100(Fe) was grown in situ on waste coconut shell-derived activated carbon (CSAC) via a straightforward solvothermal strategy (Scheme 1), producing a biomass-MOF composite with enhanced structural integrity. The effects of adsorbent dosage, contact time, pH, coexisting anions, and cycling stability were systematically evaluated. Comprehensive characterization and adsorption modeling were employed to elucidate the underlying removal mechanism. The results provide practical insights into MOF-based materials for MP remediation.

## 2. Experimental Section/Methods

### 2.1. Synthesis Procedure

Fresh coconuts sourced from Hainan Province were manually processed to remove the outer husk, middle fibrous layer, and endosperm, retaining only the inner shell. The obtained shells were thoroughly rinsed with deionized water (DI) to eliminate residual impurities and dried completely for subsequent treatment (hereafter referred to as coconut shell). A mixed activating solution composed of saturated KOH and saturated K_2_CO_3_ (1:1, *v*/*v*) [16] was prepared. The dried coconut shells were immersed in this alkaline solution for 30 min to promote chemical activation. After impregnation, the samples were dried at 60 °C and then subjected to carbonization in a tubular furnace (Model: OTF-1200X, Hefei Kejing Materials Technology Co., Ltd., China) under an argon atmosphere at 800 °C for 2 h, yielding CSAC (Coconut shell-derived activated carbon) [17].

For composite fabrication, 0.1 mol of Iron(II) chloride (FeCl_2_) was dissolved in 50 mL of 0.1 mol/L dilute hydrochloric acid (HCl) to obtain a clear precursor solution (solution A). Subsequently, 0.1 mol of CSAC was introduced and magnetically stirred for 48 h to ensure sufficient metal ion adsorption onto the C surface. Thereafter, 0.1 mol of Terephthalic acid (H_2_BDC) was added and stirred for 1 h to form a homogeneous suspension. The mixture was transferred into a polytetrafluoroethylene (PTFE)-lined autoclave and heated at 200 °C for 2 h to induce in situ crystallization of MIL-100(Fe). The resulting solid was repeatedly washed with N,N-dimethylformamide (DMF) and Absolute ethanol (C_2_H_6_O) to remove unreacted species, followed by drying to obtain the CSAC@MIL-100(Fe) composite [18].

Higher MIL-100(Fe) loadings were achieved by proportionally increasing the amounts of FeCl_2_ and H_2_BDC. MIL-100(Fe) monomers were synthesized under identical solvothermal conditions in the absence of CSAC.

#### 2.1.1. Materials

Waste coconut shells used as the C precursor were collected from roadside sources in Hainan Province, China. All reagents were of analytical grade and used as received without further purification. Iron(II) chloride (FeCl_2_) and N,N-Dimethylformamide (DMF) were purchased from Shanghai Aladdin Biochemical Technology Co., Ltd. (Haikou, China). Terephthalic acid (H_2_BDC) was supplied by Shandong Keyuan Biochemical Co., Ltd. (Haikou, China). Absolute ethanol (C_2_H_6_O), Anhydrous potassium carbonate (K_2_CO_3_), and Potassium hydroxide (KOH) were obtained from Xilong Scientific Co., Ltd. (Haikou, China). Monodisperse polystyrene microspheres (PS microspheres) were provided by Zhongke Keyou (Haikou, China).

#### 2.1.2. Characterization Techniques

The structural and physicochemical properties of the prepared materials were systematically investigated using multiple analytical methods. Surface morphology and elemental distribution were examined by field-emission scanning electron microscopy (FE-SEM) (Model: Apreo 2S+, Manufacturer: Thermo Fisher Scientific, USA), atomic force microscopy (AFM) (Model: Jupiter XR, Manufacturer: Oxford Instruments, USA), and energy-dispersive X-ray spectroscopy (EDS) (Model: OXFORD Ultim Max 65, Manufacturer: Oxford Instruments, UK). Textural parameters, including specific surface area, pore volume, and pore size distribution, were determined from N_2_ adsorption–desorption isotherms obtained using an automated surface area and porosity analyzer (Model: ASAP 2460, Manufacturer: Micromeritics Instrument Corporation, USA). Prior to analysis, all samples were degassed at 120 °C for 12 h. Crystalline phases and structural integrity were identified by X-ray diffraction (XRD) (Model: D&Advance, Manufacturer: Bruker, Germany) over a 2θ range of 0–60° at a scanning rate of 1°/min. Surface elemental composition and chemical states were analyzed by X-ray photoelectron spectroscopy (XPS) (Model: Nexsa G2, Manufacturer: Thermo Fisher Scientific, UK) for pristine materials, composites, and samples after PS adsorption. The hydrodynamic diameter and zeta potential of PS microspheres were measured using dynamic light scattering (DLS) (Model: Malvern MS3000+, Manufacturer: Malvern Panalytical, UK). Thermal behavior and decomposition characteristics were evaluated by thermogravimetric analysis (TGA) (Model: NETZSCH STA449 F5, Manufacturer: NETZSCH, Germany) under an argon atmosphere from room temperature to 800 °C at a heating rate of 5 °C/min. Functional groups were identified by Fourier transform infrared spectroscopy (FTIR) (Model: Nicolet iS5C, Manufacturer: Thermo Fisher Scientific, USA). The residual concentration of PS in adsorption experiments was quantified by ultraviolet-visible (UV-Vis) (Model: UV-3300, Manufacturer: Shanghai Mapada Instruments Co., Ltd., China) spectrophotometry.

### 2.2. Adsorption Experiments

To assess the adsorption performance of the prepared composite toward PS NP spheres, a series of batch experiments was conducted under systematically varied conditions. The influence of MIL-100(Fe) loading on the CSAC surface, adsorbent dosage, solution pH, and competitive adsorption effects was examined. PS concentrations were quantified using UV-Vis spectrophotometry. The relationship between absorbance and concentration was established through a calibration curve (Figure S1). The removal efficiency (η, %) and equilibrium adsorption capacity ($q_{e}$, mg/g) of CSAC@MIL-100(Fe) toward PS were calculated through Equations (1) and (2):

(1) $$\eta =\frac{C_{0}-C_{t}}{C_{0}}\times 100\%$$

(2) $$q_{e}=(C_{0}-C_{t})\times \frac{V}{W}$$

where $C_{0}$ (mg/L) denotes the initial concentration value of the PS solution; $C_{t}$ (mg/L) denotes the concentration value of the PS solution at time t (min); V (L) denotes the volume of added PS solution; and W (g) denotes the mass of the added material.

#### 2.2.1. Effect of Adsorbent Dosage on Adsorption Performance

The effect of adsorbent dosage on PS removal was examined using six parallel batch systems. Each system contained 70 mL of PS suspension (0.1 mg/mL), to which CSAC@MIL-100(Fe)_0.6_ was added at 5, 10, 15, 20, 25, and 30 mg, respectively. The mixtures were agitated at 400 rpm for 1200 min to reach adsorption equilibrium. After separation of the solid phase, the residual PS concentration in the supernatant was quantified.

#### 2.2.2. Effect of MIL-100(Fe) Loading on Adsorption Performance

To evaluate the role of MIL-100(Fe) loading, adsorption tests were performed under identical conditions using 20 mg of different materials (CSAC@MIL-100(Fe)_0.4_, CSAC@MIL-100(Fe)_0.5_, CSAC@MIL-100(Fe)_0.6_, and CSAC@MIL-100(Fe)_0.8_). Each adsorbent was dispersed in 70 mL of PS suspension (0.1 mg/mL) and stirred at 400 rpm for 1200 min. After equilibrium, the remaining PS concentration was determined.

#### 2.2.3. Effect of Solution pH on Adsorption Performance

The effect of pH was investigated over 2–12. Six PS suspensions (70 mL, 0.1 mg/mL) were adjusted to pH 2, 4, 6, 8, 10, and 12 using 1 mol/L HCl or KOH. Each system received 20 mg of CSAC@MIL-100(Fe)_0.6_ and was stirred at 400 rpm for 1200 min. Supernatants were subsequently analyzed to determine equilibrium PS concentrations.

#### 2.2.4. Effect of Temperature on Adsorption Performance

Adsorption behavior at different temperatures was assessed at 30, 40, 50, and 60 °C. PS suspensions (70 mL, 0.1 mg/mL) were thermostatically maintained at the designated temperatures before adding 20 mg of CSAC@MIL-100(Fe)_0.6_. After 1200 min of agitation at 400 rpm, residual PS concentrations were measured.

#### 2.2.5. Reusability Evaluation

Recyclability was assessed through seven consecutive adsorption–desorption cycles. In each cycle, 20 mg of CSAC@MIL-100(Fe)_0.6_ was contacted with 70 mL of PS suspension (0.1 mg/mL) for 1200 min. After adsorption, the material was recovered, washed with C_2_H_6_O and DI, dried, and reused in the subsequent cycle. The η of PS was determined after each cycle.

#### 2.2.6. Competitive Adsorption Cycling Experiment

To evaluate adsorption selectivity, a binary solution containing PS and methylene blue (MB) (0.1 mg/mL each, 1:1 volume ratio) was prepared. The adsorbent (20 mg) was added and stirred to equilibrium. After separation, concentrations of both PS and MB were measured. The regenerated adsorbent was reused for six successive cycles following the same washing protocol, and η were calculated accordingly.

#### 2.2.7. Effect of Adsorbent Dosage on Adsorption Performance in Seawater (Synthetic Seawater)

To simulate complex environmental conditions, PS suspensions were prepared using seawater (Using seawater with a pH of 8.1 as the solvent, prepare 70 mL of the solution at a concentration of 0.1 mg/mL). CSAC@MIL-100(Fe)_0.6_ was added at 5, 10, 15, 20, 25, and 30 mg under identical stirring conditions (400 rpm, 1200 min). The remaining PS concentration was determined after equilibrium.

#### 2.2.8. Cyclic Performance in Seawater (Synthetic Seawater)

To assess the recyclability of CSAC@MIL-100(Fe) under complex aqueous conditions, cyclic adsorption experiments were conducted in a seawater matrix. Seven batches of PS suspension (70 mL, 0.1 mg/mL) were prepared using seawater (Seawater with pH 8.1 was used as the solvent). In the first cycle, 20 mg of CSAC@MIL-100(Fe)_0.6_ was introduced into the suspension and agitated at 400 rpm for 1200 min. Following adsorption, the composite was separated from the solution, thoroughly washed with C_2_H_6_O and DI to remove residual, and dried before reuse. The supernatant from each cycle was analyzed to determine the residual PS concentration. This adsorption–desorption procedure was repeated for seven consecutive cycles, and the corresponding absorbance data were recorded.

### 2.3. Recyclability and Stability of Adsorbent

#### 2.3.1. Verification of Linear Relationship Between UV Absorbance and Solution Concentration

To establish quantitative reliability for PS determination, the linear correlation between UV-Vis absorbance and solution concentration was first validated. A series of PS MP suspensions was prepared by diluting stock solutions with DI to concentrations ranging from 15 to 100 mg/L. Absorbance measurements were recorded at 250 nm using a UV-Vis spectrophotometer (Model: UV-3300, Manufacturer: Shanghai Mapada Instruments Co., Ltd., China).

#### 2.3.2. Adsorption Mechanism Investigation Experiments

- Adsorption kinetics: Kinetic experiments were conducted to evaluate the time-dependent adsorption behavior of CSAC@MIL-100(Fe)_0.6_. PS suspensions (70 mL) with initial concentrations of 0.1, 0.3, 0.5, 0.7, and 0.9 mg/mL were prepared, and 20 mg of adsorbent was added to each system. The mixtures were agitated at 400 rpm, and sampling was performed at predetermined intervals (2, 10, 30, 120, 210, 390, 570, 900, and 1200 min). At each time point, aliquots of the supernatant were withdrawn, and residual PS concentrations were determined.
- Adsorption isotherms: Equilibrium adsorption behavior was investigated using PS suspensions (70 mL) with initial concentrations of 0.1, 0.2, 0.3, 0.4, 0.5, 0.6, 0.7, 0.8, 0.9, and 1 mg/mL. For each concentration, 20 mg of CSAC@MIL-100(Fe)_0.6_ was introduced and stirred at 400 rpm for 1200 min. After solid–liquid separation, the equilibrium concentration of PS in the supernatant was measured.

## 3. Results and Discussion

### 3.1. Material Structure Analysis

**Figure 1:**
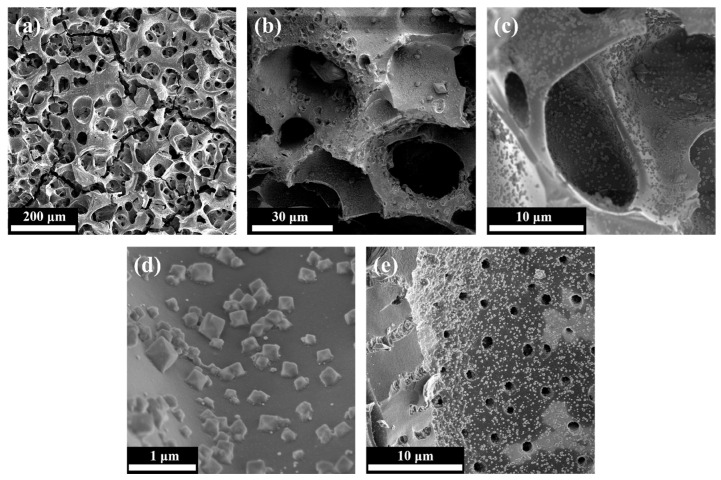
SEM images of CSAC@MIL-100(Fe): (**a**) Surface morphology of CSAC@MIL-100(Fe) at 100× magnification; (**b**–**d**) Progressive magnification images (1000×, 3500×, and 25,000×) showing the in situ growth of MIL-100(Fe) on CSAC surface; (**e**) Cross-sectional SEM image illustrating internal distribution of MIL-100(Fe) within composite.

MIL-100(Fe) was successfully grown in situ on the CSAC substrate to increase the density of accessible surface binding sites and thereby enhance adsorption performance. The morphology and interfacial integration of the composite were examined using SEM (Figure 1). The pristine CSAC framework exhibits a well-developed porous architecture with large pore openings and a rough surface texture (Figure 1a). This hierarchical structure provides not only sufficient spatial accommodation for MP capture but also abundant nucleation sites for MOF crystallization. After solvothermal treatment, discrete MIL-100(Fe) crystals are observed on the C surface (Figure 1b), displaying a relatively uniform size distribution and maintaining the characteristic octahedral morphology in Nivetha et al. [19]. In Figure 1d, MIL-100(Fe) crystals are firmly anchored to the CSAC framework rather than loosely dispersed on the surface, confirming successful in situ growth. The C matrix functions as a structural backbone, stabilizing the otherwise highly dispersible MOF microcrystals and converting them into a readily recoverable composite adsorbent. This strong interfacial integration suppresses particle aggregation in aqueous environments and enhances operational stability, thereby achieving the intended design objective of improved durability and recyclability. To further facilitate material handling and recovery, the composite was fabricated in a monolithic configuration. Cross-sectional SEM analysis demonstrates that MIL-100(Fe) is uniformly distributed throughout the internal regions of the bulk structure (Figure 1e), consistent with the surface morphology. The homogeneous growth within both external and internal domains confirms effective infiltration and anchoring of the MOF phase across the entire C scaffold.

**Figure 2:**
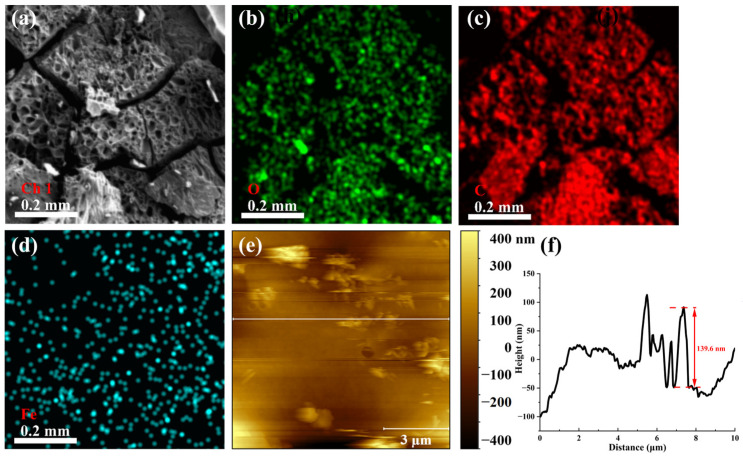
Elemental distribution and surface topography of CSAC@MIL-100(Fe): (**a**) SEM image of surface morphology; corresponding EDS elemental mappings of (**b**) O, (**c**) C, and (**d**) Fe; (**e**) AFM image; (**f**) AFM height profile analysis showing lateral dimensions of CSAC and MIL-100(Fe) particles.

Elemental mapping obtained from EDS confirms the homogeneous distribution of C, O, and Fe throughout the composite structure (Figure 2b–d). No localized enrichment or depletion of Fe species is observed, suggesting that MIL-100(Fe) crystals are evenly dispersed rather than aggregated in specific regions. This uniform elemental distribution minimizes the risk of uneven adsorption performance arising from excessive MOF stacking or insufficient growth in certain areas. The results therefore support the effectiveness of the solvothermal in situ growth strategy. The O signal is primarily associated with C–O and C=O bonding environments (Figure 2b). C originates mainly from the CSAC matrix (Figure 2c) with an additional contribution from H_2_BDC organic ligand incorporated during MIL-100(Fe) synthesis, while Fe, as the central metal node of MIL-100(Fe), exhibits consistent spatial distribution across the C surface. The co-localization of Fe with C and O further confirms successful integration of the MOF phase onto the CSAC scaffold.

AFM (Figure 2e,f) provides complementary topographical insight. The CSAC surface exhibits pronounced roughness, and discrete MIL-100(Fe) particles are clearly distinguishable. Height profile analysis indicates an interfacial dimension of approximately 500 nm, comparable to the particle size of MIL-100(Fe).

Collectively, EDS and AFM analyses corroborate the uniform growth of MIL-100(Fe) on CSAC. The well-dispersed MOF phase introduces additional accessible active sites, which is expected to substantially enhance the adsorption performance relative to pristine CSAC.

**Figure 3:**
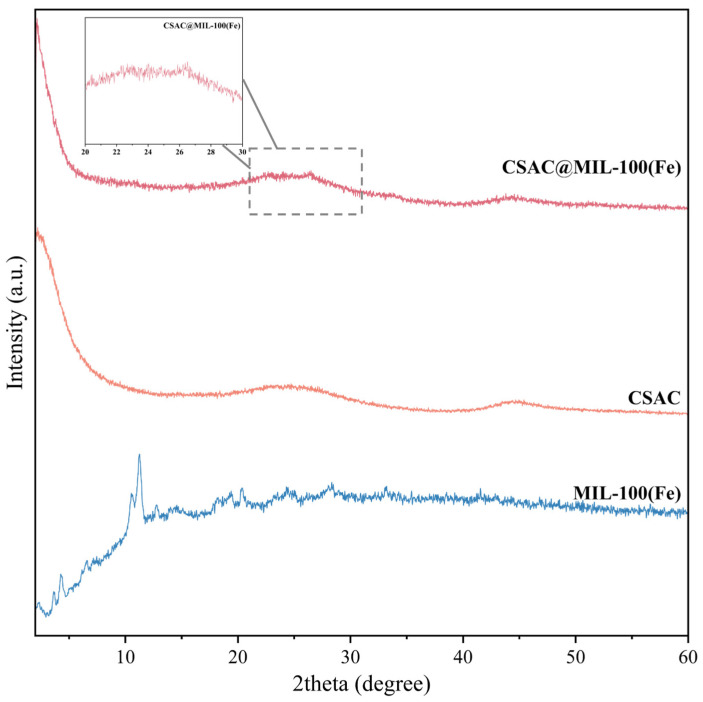
XRD patterns of MIL-100(Fe), CSAC, and CSAC@MIL-100(Fe).

Figure 3 illustrates the XRD patterns of MIL-100(Fe), pristine CSAC, and the CSAC@MIL-100(Fe) composite. The diffraction profile of MIL-100(Fe) is characterized by distinct reflections at 2θ = 3.7, 4.3, 6.2, 6.6, 10.6, and 11.3°, accompanied by a group of peaks within 17.5–20.5° and several broader reflections spanning 24.3–41.5°. These features are consistent with previously reported crystalline signatures of MIL-100(Fe) [20]. In contrast, CSAC exhibits two broad diffraction bands centered at approximately 24.8 and 44.7°, corresponding to the (002) and (100) planes of amorphous C, respectively [21]. The absence of sharp peaks indicates its predominantly disordered graphitic structure. or the CSAC@MIL-100(Fe) composite, the characteristic amorphous C humps remain evident, while additional broad reflections emerge at 2θ = 22.6, 26.5, 33.9, and 41.5°, aligning with the higher-angle diffraction features of MIL-100(Fe). Despite the reduced intensity of low-angle peaks, the presence of MOF-related diffraction signals confirms the successful in situ growth of MIL-100(Fe) on the CSAC framework without disrupting the underlying C structure.

**Figure 4:**
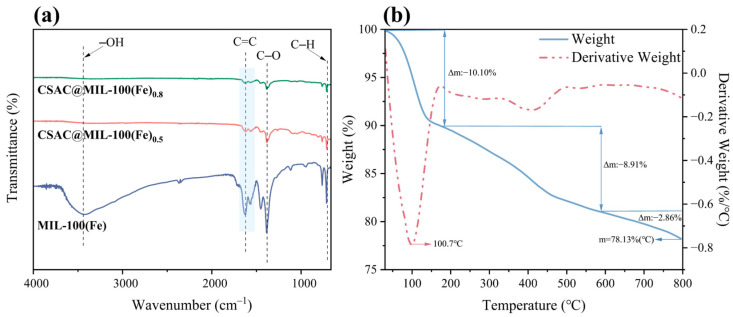
(**a**) Fourier-transform infrared (FTIR) spectra of MIL-100(Fe), CSAC@MIL-100(Fe)_0.5_, and CSAC@MIL-100(Fe)_0.8_; (**b**) TG and DTG curves of CSAC@MIL-100(Fe)_0.6_ before and after adsorption of PS MPs.

The FTIR spectrum of pristine MIL-100(Fe) (Figure 4a) exhibits characteristic vibrational features associated with its organic ligand framework and metal coordination environment. A broad band centered at 3436 cm^−1^ corresponds to O–H stretching vibrations originating from coordinated or adsorbed -OH. Bands observed at 1629, 1560, and 1449 cm^−1^ are attributed to C=C skeletal vibrations of the aromatic ligand and the asymmetric and symmetric stretching modes of –COO^−^. The signal at 1383 cm^−1^ arises from C–O stretching or C–H bending vibrations within the ligand structure. Peaks located at 1114 and 943 cm^−1^ are assigned to framework-related vibrations, including possible Fe–O stretching modes, while those at 761 and 711 cm^−1^ correspond to out-of-plane bending of aromatic C–H [22]. After integration with the CSAC substrate, noticeable spectral changes occur. The bands at 3436, 1114, and 943 cm^−1^ are significantly weakened or absent, and the –COO^−^-related peaks shift from 1629 to 1449 cm^−1^ toward lower wavenumbers (1618 and 1600 cm^−1^) with reduced intensity. The band at 1382 cm^−1^ is retained after compositing, albeit with reduced intensity, while the peaks at 761 and 710 cm^−1^ remain at nearly identical positions but also exhibit weakened signals [23]. The attenuation of these characteristic ligand vibrations suggests modification of the local bonding environment during composite formation. Such changes may arise from partial electronic interaction between the terephthalate ligands of MIL-100(Fe) and the C substrate, potentially involving π–π stacking or interfacial charge redistribution, rather than complete structural degradation. The diminished intensity or disappearance of Fe–O-related vibrational features indicates perturbation of the coordination environment surrounding the metal centers, reflecting interfacial coupling between MIL-100(Fe) and CSAC. In contrast, the persistence of the aromatic C–H out-of-plane bending vibrations demonstrates that the fundamental aromatic framework of the ligand remains largely intact after compositing.

The thermogravimetric (TG) and derivative thermogravimetric (DTG) curves of CSAC@MIL-100(Fe) after PS adsorption (Figure 4b) reveal a multi-stage weight-loss process indicative of sequential thermal events. An initial mass reduction of approximately 10.10% occurs below 200 °C, with a pronounced DTG peak centered at 100.7 °C (−0.75%/°C). This stage is primarily attributed to the removal of physically adsorbed water and residual volatile species. Owing to its highly porous structure, MIL-100(Fe) readily retains moisture from the ambient environment. In addition, trace solvent residues or low-boiling impurities associated with PS or the C matrix may contribute to this early mass loss [24]. A second weight-loss step, amounting to approximately 8.91%, is observed near 590 °C. This transition corresponds to the thermal decomposition of adsorbed PS MPs and the onset of partial degradation of the MIL-100(Fe) organic framework. Pyrolysis of PS produces volatile aromatic species such as styrene monomers, while decomposition of the MOF ligand releases gaseous products including CO_2_. Upon further heating to 800 °C, an additional 2.86% mass reduction occurs, leaving a final residue of 78.13%. This stage is attributed to gradual C matrix oxidation and the formation of thermally stable inorganic residues, including FeO_x_ and carbonaceous ash [25,26,27,28]. The DTG profile exhibits a maximum mass-loss rate at 100.7 °C (−0.75%/°C), confirming that the initial weight reduction stage is primarily governed by rapid desorption of physically adsorbed moisture. Considering the substantial overlap between the thermal decomposition temperatures of PS MPs and the MIL-100(Fe) framework, direct high-temperature regeneration could compromise the structural stability of the composite. To avoid potential framework degradation, a solvent-assisted regeneration strategy was adopted. Specifically, C_2_H_6_O washing was employed to desorb PS spheres from the CSAC@MIL-100(Fe) surface, thereby minimizing reliance on thermal treatment and preserving the coordination structure of the MOF phase.

The desorbed MPs were subsequently collected from the C_2_H_6_O phase, followed by solvent removal through drying and, if necessary, independent heat treatment. This approach effectively reduces the risk of structural collapse of MIL-100(Fe) while enabling efficient recovery of both the adsorbent and the removed MPs.

To elucidate the microstructural and electronic effects induced by varying MIL-100(Fe) loadings, XPS analysis was performed on pristine CSAC and composites with loading ratios of 0.4, 0.5, 0.6, and 0.8.

**Figure 5:**
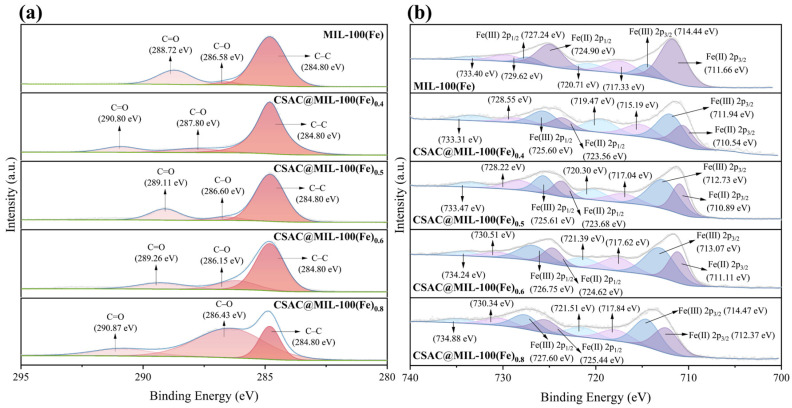
XPS spectra of MIL-100(Fe) and CSAC@MIL-100(Fe) composites with different loadings (0.4, 0.5, 0.6, and 0.8): (**a**) C 1s spectra and (**b**) Fe 2p spectra.

In Figure 5a, progressive variation in MIL-100(Fe) loading (0.4–0.8) induces noticeable changes in the C 1s spectra of CSAC@MIL-100(Fe). At a loading of 0.4, three principal components are identified at approximately 284.8 eV (C–C/C=C), 286.0 eV (C–O), and 287.5 eV (C=O). The dominant C–C signal corresponds to the graphitic carbon (GC) backbone of CSAC, while the C–O and C=O contributions indicate the presence of O-containing functional groups on the surface. With increasing MIL-100(Fe) content up to 0.8, the relative intensity of the C–O component becomes progressively enhanced. This trend suggests strengthened interfacial interactions between the MOF phase and the C substrate, accompanied by modification of the surface chemical environment. The increase in O-containing species may arise from electronic coupling or partial charge redistribution at the MOF–C interface, which can reduce local electron density and promote surface oxidation [29]. Such spectral evolution implies that higher MIL-100(Fe) loadings exert a measurable influence on the electronic structure of the CSAC surface.

A comparative analysis of the C 1s spectra at varying MIL-100(Fe) loadings reveals subtle but consistent peak shifts. As the MOF content increases, both the C–C and C–O components gradually shift toward higher binding energies. Such positive shifts suggest a decrease in electron density on the C surface, likely arising from interfacial charge redistribution between the MIL-100(Fe) phase and the CSAC substrate.

The Fe 2p spectra (Figure 5b) reveal that the oxidation state and electronic environment of iron vary with MIL-100(Fe) loading. For the low-loading sample (0.4), the Fe 2p_3/2_ and Fe 2p_1/2_ peaks located at approximately 710.5 and 724.0 eV are characteristic of Fe^3+^ species, consistent with the high-valence iron centers in MIL-100(Fe). The absence of additional satellite features or lower-binding-energy components suggests that iron predominantly remains in a stable Fe(III) state at this loading. As the MIL-100(Fe) content increases to 0.8, a slight shift in the Fe 2p_3/2_ peak toward lower binding energy is observed, accompanied by changes in peak intensity and noticeable peak broadening. The emergence of lower-binding-energy contributions indicates partial reduction of Fe^3+^ to Fe^2+^, implying modification of the local coordination environment. Such spectral evolution suggests enhanced electronic interaction between the MIL-100(Fe) phase and the CSAC substrate at higher loadings [30,31]. In addition to binding energy shifts, the relative proportions of Fe^2+^ and Fe^3+^ species vary with increasing loading, and the progressive broadening of the Fe 2p_3/2_ envelope reflects increased electronic heterogeneity. This broadening may arise from interfacial charge transfer, lattice distortion, or variation in coordination symmetry induced by stronger MOF–C coupling [32].

**Figure 6:**
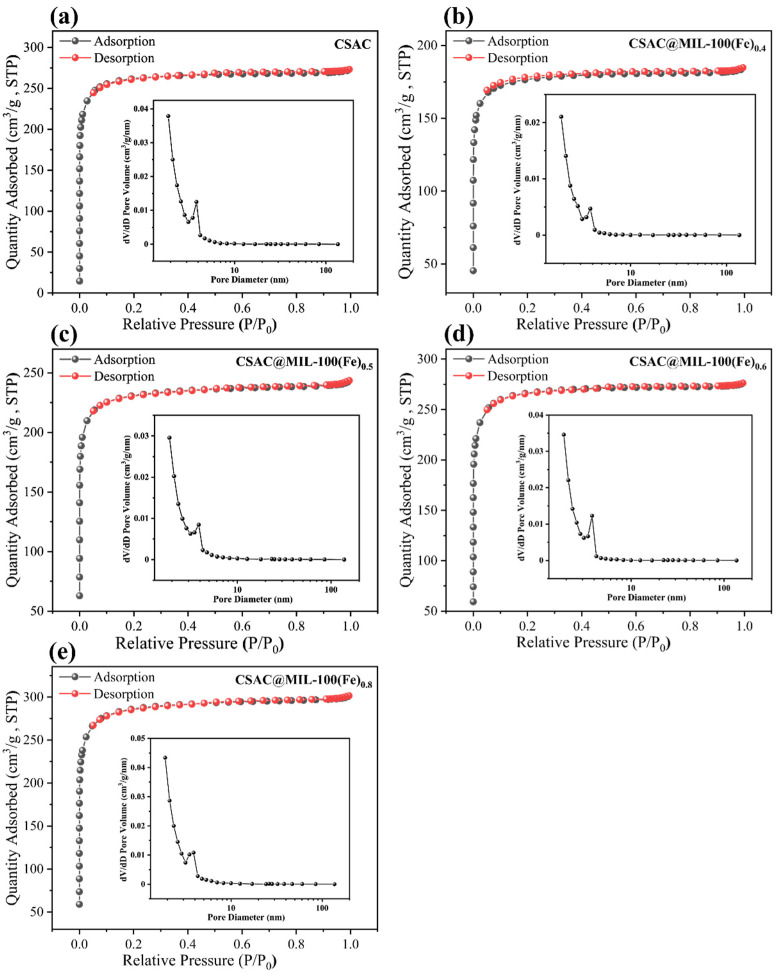
BET and pore size distribution analysis: (**a**) CSAC; (**b**) CSAC@MIL-100(Fe)_0.4_; (**c**) CSAC@MIL-100(Fe)_0.5_; (**d**) CSAC@MIL-100(Fe)_0.6_; (**e**) CSAC@MIL-100(Fe)_0.8_.

Figure 6a–e depicts the N_2_ adsorption–desorption isotherms and corresponding pore size distributions of pristine CSAC and composites with different MIL-100(Fe) loadings. All samples exhibit typical Type I isotherms, characteristic of predominantly microporous materials [33], which is consistent with the Brunauer–Emmett–Teller (BET)-derived pore parameters summarized in Table 1. The pristine CSAC shows a high specific surface area of 1034.22 m^2^/g and a pore volume of 0.4224 cm^3^/g, with an average pore diameter of approximately 1.62 nm, confirming its microporous nature. Upon introducing MIL-100(Fe)_0.4_, the BET surface area decreases markedly to 699.86 m^2^/g and the pore volume to 0.2858 cm^3^/g. This reduction suggests partial blockage of intrinsic C micropores by initially deposited MOF particles. With further increases in MIL-100(Fe) loading (0.5–0.8), both the specific surface area and pore volume progressively recover and eventually exceed those of pristine CSAC. At a loading of 0.8, the surface area reaches 1127.68 m^2^/g and the pore volume of 0.3766–0.4662 cm^3^/g. The average pore diameter remained nearly constant at approximately 1.6 nm across all samples, indicating that incorporation of MIL-100(Fe) primarily influences pore volume and specific surface area rather than altering the intrinsic pore size distribution. At low MIL-100(Fe) loadings, partial coverage of the C surface by newly formed MOF particles leads to blockage of some native micropores, resulting in a noticeable decrease in specific surface area. With further increases in MIL-100(Fe) content, however, the intrinsic porosity of the MOF phase and the formation of interparticle voids compensate for the initial surface-area loss. Consequently, the overall specific surface area recovers and ultimately surpasses that of pristine CSAC. The increased surface roughness and additional interstitial spaces introduced by MIL-100(Fe) crystals further enhance external porosity, improving accessibility of adsorption sites. Therefore, higher MIL-100(Fe) loadings provide a synergistic structural effect: while minor pore obstruction may occur at low loading, increased MOF content introduces abundant coordination-active sites and supplementary porous domains. This combination yields a composite with high surface area, rich active-site density, and a hierarchical pore structure advantageous for PS capture. For adsorption experiments, commercially available monodisperse PS microspheres with a nominal diameter of 500 nm were used without further modification. Given that the diameter of the PS microspheres (~500 nm) is several orders of magnitude larger than the average pore size of the composite (1.6 nm), adsorption cannot occur through pore-filling within the microporous framework. Instead, PS capture is primarily governed by interactions occurring on the external surface of the adsorbent and within interparticle voids or macroporous channels. The incorporation of MIL-100(Fe) effectively modulates the textural properties of the composite. While the average pore diameter remains essentially unchanged at approximately 1.6 nm, both the specific surface area and pore volume exhibit a loading-dependent evolution, initially decreasing due to partial pore blockage and subsequently increasing as the intrinsic porosity of MIL-100(Fe) becomes dominant. At a loading level of 0.6, the composite achieves a favorable balance between structural accessibility and active-site density, exhibiting a high specific surface area (1050.44 m^2^/g) together with abundant coordination-active sites. This optimized structural configuration is therefore expected to provide superior adsorption performance toward PS MPs.

### 3.2. MP Adsorption Experiment

#### 3.2.1. Effect of Adsorbent Dosage

**Figure 7:**
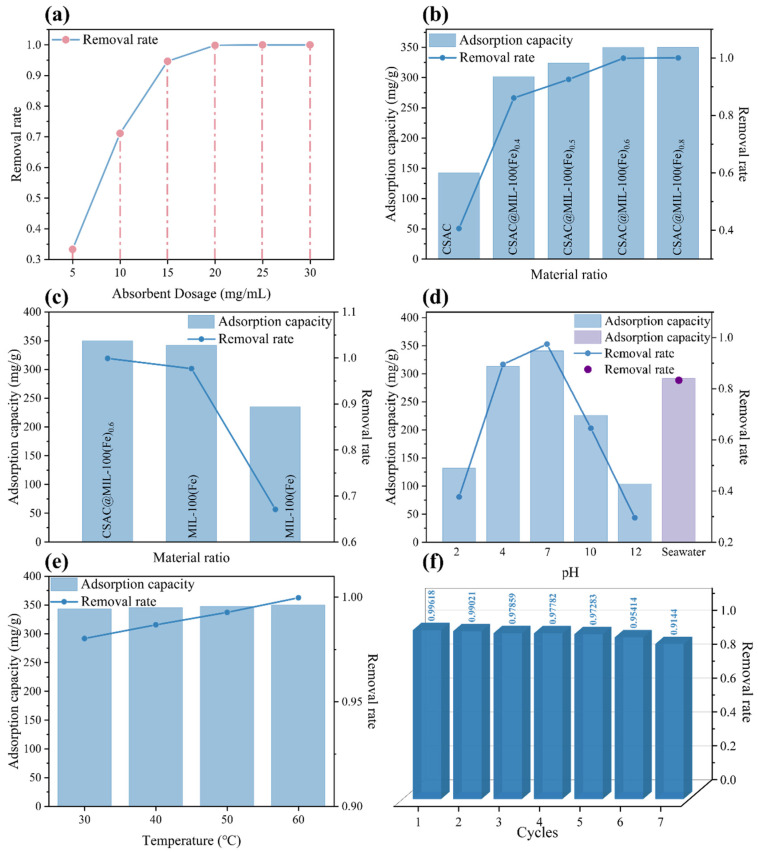
Adsorption performance of CSAC@MIL-100(Fe): (**a**) Effect of adsorbent dosage on η for PS using CSAC@MIL-100(Fe)_0.6_; (**b**) Comparison of $q_{e}$ and η of CSAC and CSAC@MIL-100(Fe) composites with different MIL-100(Fe) loadings (0.4–0.8); (**c**) Comparison of adsorption performance using 20 mg CSAC@MIL-100(Fe)_0.6_, 20 mg MIL-100(Fe), and an equivalent mass (12 mg) of MIL-100(Fe); (**d**) Effect of solution pH; (**e**) Effect of temperature; (**f**) Recyclability of CSAC@MIL-100(Fe)_0.6_.

Figure 7a presents the effect of adsorbent dosage on η of PS. Under an initial PS concentration of 0.1 mg/mL, increasing the dosage of CSAC@MIL-100(Fe)_0.6_ from 5 to 30 mg results in a steady rise in η, from 35.8 to 98.2%. Notably, the improvement becomes less pronounced when the dosage exceeds 20 mg. Beyond this threshold, the η approaches saturation, and further increases in adsorbent mass yield only marginal gains. Such behavior can be attributed to the near-complete utilization of accessible PS particles in solution; once the concentration of adsorption sites surpasses the available adsorbate molecules, additional sites remain unoccupied. Furthermore, excessive solid content may lead to particle aggregation or overlap of active surfaces, reducing the η [15]. Considering both adsorption efficiency and material economy, 20 mg was selected as the optimal dosage for subsequent experiments, as it achieves near-maximal removal while avoiding unnecessary material consumption.

#### 3.2.2. Effect of MIL-100(Fe) Loading

Based on the dosage optimization results (Figure 7a), the initial PS concentration was fixed at 0.1 mg/mL and the adsorbent dosage at 20 mg for subsequent loading-dependent experiments. Under these standardized conditions, the MIL-100(Fe) content was systematically varied to determine the optimal composite composition. In Figure 7b, the $q_{e}$ increases with increasing MIL-100(Fe) loading up to 0.6, after which the improvement becomes negligible. The sample with a loading ratio of 0.6 exhibits the highest $q_{e}$, while further increases to 0.8 yield comparable but not significantly enhanced performance. This evolution closely parallels the BET results, confirming that adsorption efficiency is strongly governed by accessible surface area and the density of active sites. At low loading (0.4), partial pore blockage and limited MOF coverage reduce the available surface area, resulting in inferior adsorption performance. In contrast, moderate loadings (0.5–0.6) achieve an optimal balance between preserving the intrinsic porosity of CSAC and introducing metal coordination sites from MIL-100(Fe). The coexistence of hydrophobic C domains and polar Fe-based coordination centers promotes a synergistic adsorption mechanism involving hydrophobic interactions and surface coordination effects [34,35]. When the loading reaches 0.8, excessive MOF accumulation likely induces particle aggregation and partial surface shielding, diminishing the marginal gain in active sites. Consequently, CSAC@MIL-100(Fe)_0.6_ is identified as the optimal configuration.

To verify the necessity of integrating MIL-100(Fe) with CSAC, control experiments were conducted using MIL-100(Fe) alone under identical experimental conditions (Figure 7c). When an equivalent mass of MIL-100(Fe) was employed as the adsorbent at the same initial PS concentration, its $q_{e}$ was measured at 341.9 mg/g, slightly lower than that of CSAC@MIL-100(Fe)_0.6_ (349.7 mg/g). The corresponding η was also marginally reduced. To ensure a rigorous comparison, the exact MIL-100(Fe) content within the CSAC@MIL-100(Fe)_0.6_ composite was quantified, and the same mass of pure MIL-100(Fe) was subsequently tested. Under these strictly equivalent conditions, both $q_{e}$ and η of standalone MIL-100(Fe) were distinctly inferior to those of the composite material. This performance enhancement can be attributed to the synergistic interaction between the porous C matrix and the MOF phase. The CSAC framework improves structural stability, prevents particle aggregation, and increases accessible adsorption interfaces, while MIL-100(Fe) introduces coordination-active sites. The composite architecture therefore maximizes site utilization efficiency and mass-transfer accessibility. These findings demonstrate that compositing MIL-100(Fe) with CSAC not only enhances adsorption performance but also improves material utilization efficiency, offering a more economically viable and structurally stable approach for practical MP remediation.

#### 3.2.3. Effect of pH on the Adsorption Properties of CSAC@MIL-100(Fe)

In real environmental systems, water pH varies considerably and rarely remains at the neutral conditions typically employed in laboratory studies. Therefore, evaluating adsorption performance across a broad pH range is essential. In Figure 7d, CSAC@MIL-100(Fe)_0.6_ maintains a η above 80% within the pH of 4–9, demonstrating substantial operational stability. The optimal performance is observed near neutral conditions (pH ≈ 7), where the η reaches 99.7%. This pH tolerance can be attributed primarily to electrostatic interactions. Zeta potential analysis (Figure S2) indicates that PS particles carry a negative surface charge, while CSAC@MIL-100(Fe)_0.6_ exhibits a positively charged surface within this pH window. The resulting electrostatic attraction facilitates efficient adsorption. When the pH exceeds 10, both $q_{e}$ and η decline markedly. Under strongly alkaline conditions, deprotonation of surface functional groups reduces the positive charge density of the adsorbent, weakening electrostatic attraction and potentially inducing repulsion between the negatively charged PS particles and the adsorbent surface. In addition, OH^−^ may compete with PS for coordination-active sites on the MIL-100(Fe) framework, further limiting adsorption. As electrostatic contributions diminish, surface complexation and non-electrostatic interactions become relatively more significant.

#### 3.2.4. Effect of Temperature

Figure 7e illustrates the influence of temperature (30–60 °C) on the adsorption performance of CSAC@MIL-100(Fe)_0.6_ toward PS MPs. A clear positive correlation between temperature and $q_{e}$ is observed within this range. As the temperature increases from 30 to 60 °C, the $q_{e}$ rises from 343.08 to 349.90 mg/g, while the η improves from 98.02 to 99.97%, approaching complete removal. The gradual enhancement in adsorption with increasing temperature suggests that the process is endothermic rather than purely governed by physical adhesion. Elevated temperature reduces solution viscosity and enhances Brownian motion of PS particles, thereby accelerating mass transfer from the bulk solution to the adsorbent surface. Improved diffusion kinetics increase the probability of effective collisions between PS spheres and active sites, contributing to higher equilibrium uptake. In addition to kinetic effects, temperature may influence the interfacial interaction strength. The MIL-100(Fe) framework possesses structural flexibility typical of certain MOFs, and mild thermal activation can facilitate access to coordination-active sites or enhance surface exposure. Moreover, adsorption likely involves multiple interaction mechanisms, including π–π stacking between aromatic rings of PS and the MOF ligands, hydrophobic interactions with the C matrix, and coordination interactions with Fe centers. The formation of such interactions requires overcoming an energetic barrier; increasing temperature provides additional activation energy, thereby favoring adsorption and shifting the equilibrium toward the adsorbed state [36,37].

#### 3.2.5. Recyclability

To enhance practical applicability and reduce operational costs, CSAC@MIL-100(Fe) was fabricated in a monolithic form to facilitate straightforward separation from aqueous systems and minimize the risk of secondary contamination. The recyclability of CSAC@MIL-100(Fe)_0.6_ was evaluated through consecutive adsorption–desorption cycles (Figure 7f). After each adsorption run, the material was recovered and subjected to solvent-assisted regeneration. Specifically, it was repeatedly washed with anhydrous C_2_H_6_O to desorb retained PS particles, followed by thorough rinsing with DI to eliminate residual solvent. The regenerated adsorbent was then oven-dried prior to reuse in the subsequent cycle. After seven cycles, the $q_{e}$ remained at 320.0 mg/g, and the η, although slightly reduced compared to the initial cycle, consistently exceeded 90%. The modest decline in performance may be attributed to partial blockage of active sites or minor structural alterations during repeated handling; however, the overall adsorption capability was largely preserved. These results demonstrate that CSAC@MIL-100(Fe)_0.6_ exhibits stable structural integrity and effective regenerability. The combination of facile recovery and sustained adsorption performance significantly lowers material consumption and operational costs, underscoring the composite’s potential for practical MP remediation applications.

#### 3.2.6. Effect of Competitive Adsorption

**Figure 8:**
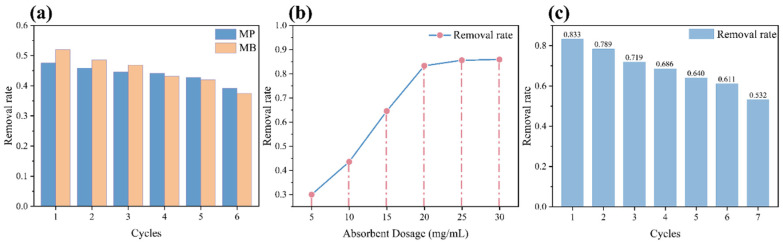
Adsorption performance of CSAC@MIL-100(Fe)_0.6_ under complex conditions: (**a**) η of PS and MB in a binary system and their evolution over six adsorption–desorption cycles; (**b**) Effect of adsorbent dosage on η of PS in a seawater matrix at identical initial concentrations; (**c**) Cyclic adsorption performance in seawater.

In the single-component system (initial PS concentration = 0.1 mg/mL), CSAC@MIL-100(Fe)_0.6_ demonstrated excellent cycling stability. The η remained above 95% during the first six cycles and retained 91.44% after the seventh cycle, confirming that the regeneration protocol effectively restores the majority of active adsorption sites and preserves structural integrity under repeated use. In contrast, markedly different behavior was observed in a binary system containing PS and Methyl Blue (MB) at equal initial concentrations (0.1 mg/mL each, 1:1 volume ratio; Figure 8a). In the first cycle, the η of PS decreased sharply to 47.55%, while MB removal reached 52.00%, indicating preferential adsorption of MB. After six cycles, the η of PS and MB declined further to 39.14 and 37.43%, respectively. The pronounced performance reduction compared with the single-component system highlights the strong competitive adsorption effect introduced by MB. The initial selectivity toward MB can be attributed to its planar conjugated structure and electron-rich functional groups, which favor π–π stacking interactions with the C matrix and coordination interactions with exposed Fe centers in MIL-100(Fe) [38,39,40]. With increasing adsorption cycles, the initial selectivity toward MB gradually diminished. By the fourth cycle, the η of PS and MB converged, indicating a progressive redistribution of accessible adsorption sites and a dynamic restructuring of the competitive adsorption regime. After six cycles, the η declined to 39.14% for PS and 37.43% for MB, corresponding to cumulative decreases of 17.9 and 28.0%, respectively. This performance deterioration can be primarily attributed to active-site occupation and pore blockage effects. In the single-component PS system, all accessible adsorption sites—including mesoporous domains and coordination-active Fe centers—are available for interaction with PS particles. In contrast, within the binary system, MB molecules, characterized by their planar conjugated structure and electron-rich functional groups, exhibit strong affinity toward the open metal sites in the MIL-100(Fe) framework. Through coordination interactions and π–π stacking with the C matrix, MB preferentially occupies high-energy adsorption sites. Such preferential occupation not only consumes energetically favorable binding centers but may also induce steric hindrance around adjacent sites, effectively reducing the number of accessible regions available for PS adsorption [41,42].

Although C_2_H_6_O washing during regeneration can remove a substantial fraction of adsorbed dye molecules, a portion of strongly bound species may remain confined within the microporous domains of the composite. Progressive accumulation of these residual species over repeated cycles alters the distribution and chemical nature of active sites on the surface, thereby reducing selective adsorption capability and decreasing the number of accessible high-energy binding sites. Consequently, the effective surface area available for subsequent adsorption gradually diminishes. The hierarchical pore architecture further contributes to performance degradation. In the early adsorption stages, the relatively smaller MB molecules can diffuse more rapidly into mesoporous regions, gaining preferential access to internal coordination sites. As cycling proceeds, partial occupation of pore channels by both PS and MB molecules generates steric congestion and diffusion barriers. In addition, competitive interactions within the binary system exacerbate performance loss. PS and MB may interfere through steric hindrance or electrostatic effects, disrupting optimal adsorption configurations and accelerating active-site depletion. Repeated drying and liquid-phase cycling may also induce localized structural fatigue, including partial distortion of the MIL-100(Fe) coordination framework or weakening of the interfacial bonding between the MOF phase and the CSAC support.

#### 3.2.7. Effect of Adsorbent Dosage in Seawater (Synthetic Seawater)

Under ideal pure-water conditions, PS removal exhibited a typical dose-dependent enhancement. As the adsorbent dosage increased from 5 to 30 mg/mL, the η rose from 35.87 to 98.25%. Notably, efficiencies exceeded 93% at 15 mg/mL, indicating that the majority of accessible active sites approached effective utilization within this dosage range. A pronounced transition occurred between 15 and 20 mg/mL, where η increased sharply, suggesting a critical threshold corresponding to near-complete coverage of available adsorption interfaces. In contrast, adsorption performance in seawater (Figure 8b) was substantially suppressed. Under identical dosage conditions, η ranged only from 29.92 to 85.89%, and a plateau emerged at higher dosages (25–30 mg/mL), reflecting strong matrix interference. The reduced adsorption performance in seawater can be primarily attributed to ionic competition and electrostatic screening effects. Seawater contains high concentrations of cations such as Na^+^, Ca^2+^, Mg^2+^, which can associate with negatively charged sites on the PS MP surface, forming an ion-screening layer. This layer partially neutralizes surface charge and weakens electrostatic interactions between PS and the positively charged regions of CSAC@MIL-100(Fe). Simultaneously, these cations may compete for coordination with the open metal sites within the MIL-100(Fe) framework, thereby decreasing the availability of high-energy adsorption sites for PS capture. In addition to ionic effects, steric obstruction within the pore network contributes to performance suppression. Dissolved organic matter and colloidal species present in seawater can adsorb onto the composite surface or partially block mesoporous channels, increasing diffusion resistance for PS particles. This pore-channel interference is particularly significant at lower adsorbent dosages, where active sites are limited and transport limitations more strongly influence η. Furthermore, the high ionic strength of seawater compresses the electrical double layer at the solid–liquid interface, a phenomenon described by Debye screening theory. The resulting reduction in electrostatic interaction range diminishes the long-range attraction between the composite and PS MPs, thereby lowering the initial adsorption driving force. The adsorption process exhibits pronounced critical behavior in the pure water system. A distinct threshold phenomenon is observed within the adsorbent dosage range of 15–20 mg/mL, where the η increases sharply from 71.28 to 97.41%. This dosage interval therefore represents the critical point at which the density of available active sites becomes sufficient to approach near-complete surface coverage of PS MPs. In contrast, this critical behavior is markedly attenuated in the seawater system. The transition point shifts toward higher dosages, and the magnitude of efficiency improvement is substantially reduced. Such a delayed and weakened response indicates that the complex ionic matrix of seawater suppresses adsorption kinetics and restricts effective site accessibility.

#### 3.2.8. Effect of Seawater (Synthetic Seawater) on Cycling Performance

In a pure water system, CSAC@MIL-100(Fe)_0.6_ demonstrated excellent cycling stability. The η reached 97.41% in the first cycle and remained at 91.81% after seven cycles, corresponding to a cumulative decay of only 5.6%. Notably, the efficiency remained above 95% during the first four cycles, indicating strong structural integrity and effective regeneration under simplified conditions. In contrast, the seawater system (Figure 8c) exhibited markedly accelerated performance deterioration. The initial η was 83.31%, which decreased sharply to 53.24% after seven cycles, yielding a cumulative decay of 36.1%. Unlike the relatively stable behavior in pure water, a continuous decline was observed from the first cycle onward in seawater, suggesting persistent matrix-induced degradation. This accelerated decay can be attributed to combined chemical and physical effects of the saline environment. High concentrations of anions such as Cl^−^ and SO_4_^2−^ may competitively coordinate with Fe centers in the MIL-100(Fe) framework, perturbing the original coordination environment and gradually weakening structural stability [43,44]. Simultaneously, the accumulation of cations such as Na^+^ and Mg^2+^ within the pore network may trigger ion-exchange processes, leading to partial and potentially irreversible occupation of coordination-active sites. The synergistic action of both anions and cations in seawater thus disrupts the original adsorption equilibrium, progressively decreasing the number of accessible high-energy binding sites. In addition to chemical competition, structural degradation of the pore framework may occur under prolonged exposure to seawater. Dissolved salts can undergo repeated crystallization–dissolution cycles within confined pore spaces, generating mechanical stress on the pore walls and inducing localized collapse or distortion of the mesoporous structure. Concurrently, colloidal particles and dissolved organic matter may deposit within the channels, forming physical diffusion barriers. The combined effects of chemical erosion and physical obstruction reduce effective pore accessibility and diminish the specific surface area available for adsorption. Moreover, irreversible alterations in interfacial chemical properties further impair adsorption performance. The high ionic strength of seawater compresses the electrical double layer at the solid–liquid interface, weakening electrostatic attraction between the adsorbent and PS MPs. More critically, specific adsorption of seawater constituents onto the composite surface can induce rearrangement of surface charge distribution and attenuate the activity of functional groups involved in adsorption [45,46]. These interfacial alterations are not readily reversible during regeneration, resulting in a gradual decline in adsorption performance over repeated cycles. In particular, the ethanol-based regeneration strategy exhibits inherent limitations under saline conditions. Strong coordination interactions formed between seawater ions and the active sites of the composite cannot be completely disrupted through simple solvent washing, leading to incomplete recovery of adsorption sites after each cycle. Furthermore, residual salts retained within the pore structure may recrystallize during the drying process, generating additional mechanical stress and exacerbating structural degradation of the mesoporous framework. This repeated crystallization-dissolution process progressively weakens pore integrity and further reduces accessible surface area. Consequently, both chemical irreversibility and structural fatigue contribute to the cumulative deterioration of adsorption efficiency in seawater environments.

### 3.3. Adsorption Mechanism Analysis

**Figure 9:**
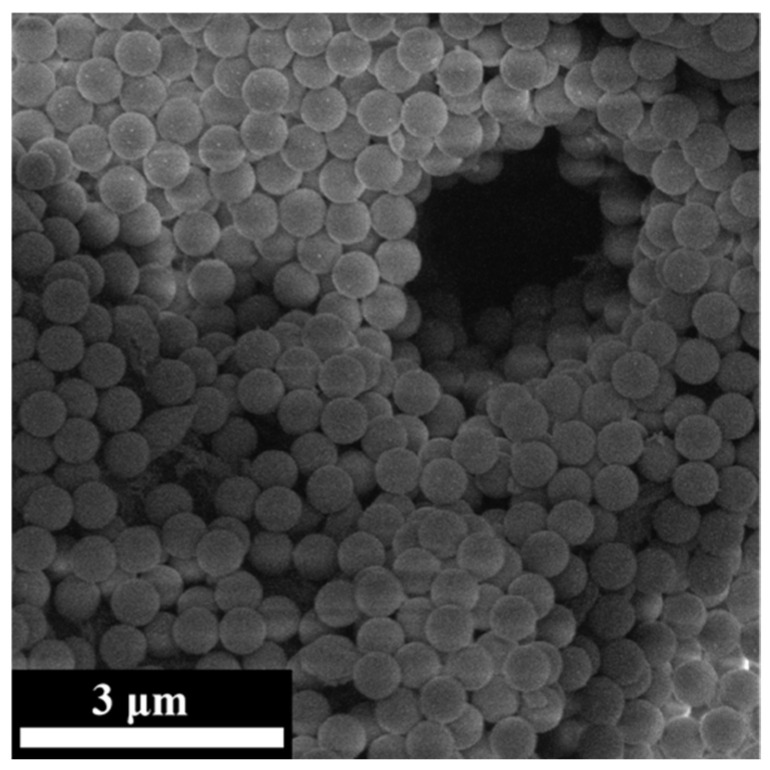
Image (10,000× magnification) showing surface morphology of CSAC@MIL-100(Fe) after adsorption of PS MPs.

To visually evaluate adsorption behavior, the CSAC@MIL-100(Fe) composite was immersed in a dispersed PS MP suspension and subsequently characterized by SEM. The post-adsorption morphology provides direct structural evidence of PS capture. In Figure 9, the surface of the composite after adsorption is densely covered with PS microspheres. The particles are uniformly distributed and appear firmly anchored within macroporous regions and interparticle voids of the composite. The extensive surface coverage effectively obscures the underlying MIL-100(Fe) crystalline features, indicating strong and stable immobilization of PS particles. The absence of loosely attached aggregates suggests that adsorption involves robust interfacial interactions rather than simple mechanical deposition. These observations confirm that the hierarchical architecture of CSAC@MIL-100(Fe) provides abundant accessible binding interfaces for PS MPs in aqueous systems.

For elucidating the adsorption mechanism at the molecular level, XPS was employed to analyze changes in surface chemical states before and after PS adsorption. In addition, adsorption kinetics and equilibrium isotherm models were fitted to the experimental data to clarify the dominant interaction pathways and rate-controlling steps governing the adsorption process.

**Figure 10:**
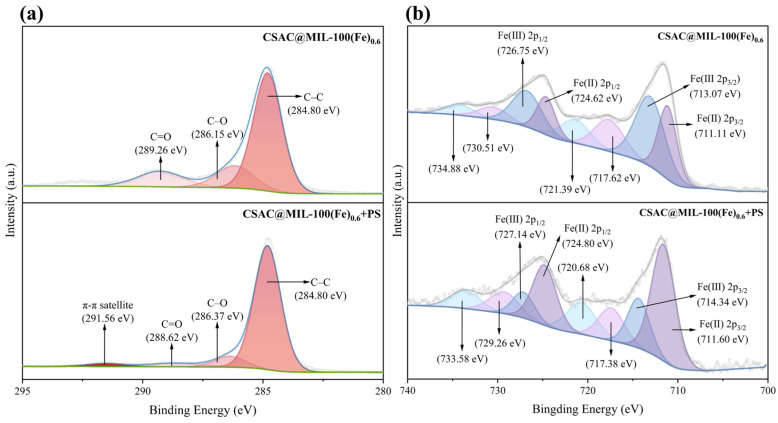
XPS spectra of CSAC@MIL-100(Fe) before and after PS MP adsorption: (**a**) C 1s spectra and (**b**) Fe 2p spectra.

To further investigate the interfacial interactions between CSAC@MIL-100(Fe)_0.6_ and PS MPs, XPS analysis was performed before and after adsorption to examine changes in elemental valence states and bonding environments. For the pristine composite, the C 1s spectrum (Figure 10a) can be deconvoluted into characteristic components at 284.8 eV (C–C/C=C), 286.15 eV (C–O), and 289.26 eV (C=O). After PS adsorption, the spectrum exhibits peaks at 284.8 eV (C–C), 286.37 eV (C–O), and 288.62 eV (C=O), along with an additional feature at 291.56 eV attributed to π–π* shake-up interactions. The decrease in binding energy of the C=O component suggests altered electronic density around O-containing groups, possibly due to H bonding or interfacial electronic interactions with PS molecules. The enhancement of the C–O signal further supports the involvement of O-containing functional groups in the adsorption process. Notably, the C–C peak area increases substantially after adsorption (from 62,605.70 to 82,248.86), reflecting the introduction of additional aromatic C from PS onto the composite surface. The π–π* transition peak at 291.56 eV provides strong evidence of π–π stacking interactions between the benzene rings of the terephthalate ligands in MIL-100(Fe) and the aromatic backbone of PS, confirming the role of conjugated interactions in stabilizing adsorption. In the Fe 2p spectrum (Figure 10b), slight shifts toward higher binding energies are observed for both Fe 2p_3/2_ and Fe 2p_1/2_ after adsorption. Such shifts indicate changes in the local electronic environment of Fe centers, suggesting interaction between PS and exposed metal sites [47].

**Scheme 2:**
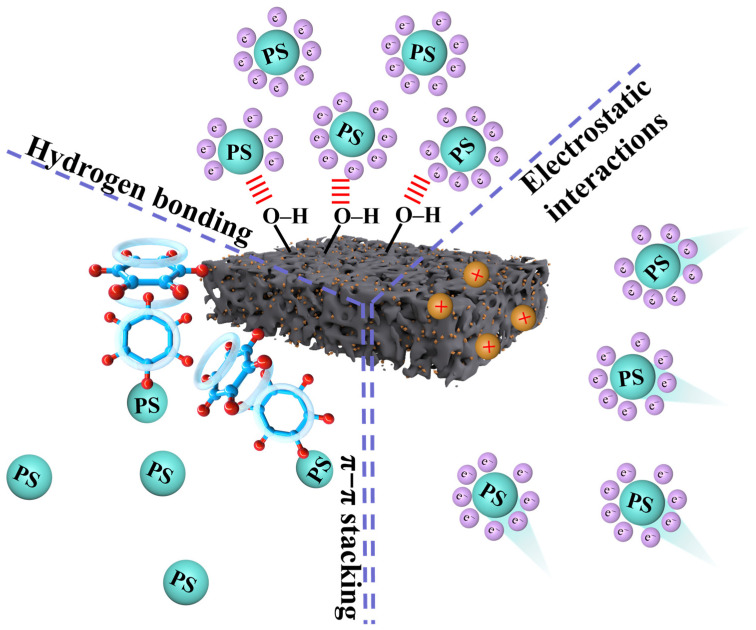
Proposed adsorption mechanism illustrating interfacial interactions between PS MP spheres and the CSAC@MIL-100(Fe) composite.

Scheme 2 illustrates the proposed adsorption mechanism of PS MPs on the CSAC@MIL-100(Fe) composite. After successful in situ growth of MIL-100(Fe) on the CSAC framework, the monolithic composite was introduced into a dilute PS suspension to enable adsorption. Owing to the substantial size of PS microspheres relative to the intrinsic micropores (1.6 nm), adsorption primarily occurs on the external surface and within interparticle voids rather than inside microporous channels. H bonding between O-containing functional groups on the composite surface and PS, π–π stacking interactions between aromatic rings of PS and the terephthalate ligands of MIL-100(Fe), and electrostatic attraction collectively contribute to particle immobilization. While micropores and narrow mesopores are largely inaccessible due to size exclusion, macropores and hierarchical void structures serve as efficient mass-transfer channels, facilitating diffusion of PS particles into the interior of the composite. The incorporation of MIL-100(Fe) within these regions introduces additional coordination-active sites, increasing the density of effective binding interfaces. The synergistic interplay between the porous C scaffold and the MOF phase—combining hydrophobic domains, coordination centers, and hierarchical transport pathways—ultimately enhances adsorption efficiency and stability.

#### 3.3.1. Adsorption Isotherms

**Figure 11:**
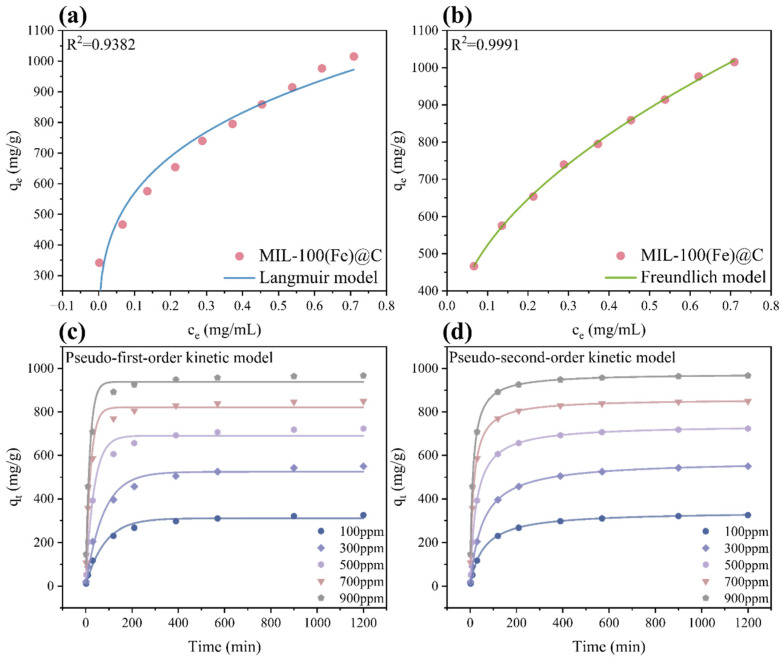
Adsorption isotherm and kinetic model fitting at 25 °C: (**a**) Langmuir isotherm; (**b**) Freundlich isotherm; (**c**) PFO kinetic model; (**d**) PSO kinetic model.

To elucidate the adsorption behavior of PS MPs on CSAC@MIL-100(Fe), the experimental data were fitted using the Langmuir (Figure 11a) and Freundlich (Figure 11b) isotherm models. Adsorption experiments were performed at pH 7 with an adsorbent dosage of 20 mg and initial PS concentrations ranging from 0.1 to 1.0 mg/mL. The corresponding fitting curves are depicted in Figure 11, and the derived parameters are summarized in Table 2. The Langmuir model (Equation (3)) assumes monolayer adsorption onto a homogeneous surface with a finite number of energetically identical sites. It further presumes that each adsorption site accommodates only one adsorbate molecule and that no interaction occurs between adsorbed species. In contrast, the Freundlich model (Equation (4)) is an empirical equation that describes adsorption on heterogeneous surfaces. The mathematical expressions of the two models are given as follows:

(3) $$q_{e}=\frac{Q_{\max}k_{L}C_{e}}{1\;+\;k_{L}C_{e}}$$

(4) $$q_{e}={\;k}_{F}C_{e}^{1/n}$$

where $C_{e}$ (mg/L) denotes the equilibrium concentration of PS residuals; $k_{F}$ ((mg^1−1/n^L^1/n^)/g) and $k_{L}$ (L/mg) denote the adsorption equilibrium constants for the Freundlich and Langmuir models, respectively; and n denotes the adsorption intensity parameter.

In Figure 11a,b, the $q_{e}$ of CSAC@MIL-100(Fe) increases progressively with rising initial PS concentration, reflecting enhanced driving force for mass transfer at higher solute concentrations. The correlation coefficients (R^2^) listed in Table 2 indicate that the Freundlich model provides a better fit to the experimental data than the Langmuir model. This suggests that PS adsorption occurs predominantly on a heterogeneous surface with non-uniform energy distribution, allowing for multilayer adsorption rather than idealized monolayer coverage. Such behavior is consistent with the composite’s hierarchical structure and the coexistence of multiple interaction sites (C domains, coordination centers, and surface functional groups). Although the Langmuir model shows comparatively lower fitting accuracy, the calculated separation factor ($R_{L}$) derived from $k_{L}$ falls within the range 0 < $R_{L}$ < 1, confirming that the adsorption process is thermodynamically favorable. The high $k_{F}$ and the n greater than 1 further indicate favorable adsorption and strong affinity between PS particles and the composite surface. A larger n reflects increased surface heterogeneity and a more pronounced curvature of the isotherm toward the $q_{e}$ axis, signifying efficient uptake at moderate concentrations. The maximum adsorption capacity ($q_{m}$) estimated from the Langmuir model reaches 746.27 mg/g, demonstrating the substantial adsorption potential of CSAC@MIL-100(Fe) for PS MPs.

#### 3.3.2. Adsorption Kinetics

To investigate the influence of contact time on PS adsorption by CSAC@MIL-100(Fe), the experimental kinetic data were fitted using the pseudo-first-order (PFO) and pseudo-second-order (PSO) models (Figure 11c,d). The corresponding kinetic parameters are summarized in Table 3, providing insight into the rate-controlling steps and the nature of the adsorption process. The PFO model (Equation (5)) assumes that the adsorption rate is proportional to the number of unoccupied adsorption sites, and it is commonly associated with diffusion-controlled or physisorption-dominated processes. In contrast, the PSO model (Equation (6)) assumes that the adsorption rate is proportional to the square of the number of unoccupied sites and is generally interpreted as indicating chemisorption involving valence forces, electron sharing, or electron exchange between the adsorbent and adsorbate. The mathematical expressions of the two kinetic models are obtained as follows:

(5) $$q_{t}=q_{e}(1-e^{-k_{1}t})$$

(6) $$q_{t\;}=\frac{q_{e}^{2}k_{2}t}{1+{k_{2}q}_{e}t}$$

where $q_{t}$ (mg/g) denotes the adsorption capacities at time t; while $k_{1}$ (min^−1^) and $k_{2}$ (g/(mg·min)) denote the kinetic rate constants for the PFO and PSO models, respectively.

In Figure 11c,d, the $q_{e}$ of CSAC@MIL-100(Fe) toward PS increases rapidly during the initial 120 min, followed by a gradual approach to equilibrium. This behavior suggests a two-stage adsorption process, consisting of a fast surface adsorption phase driven by abundant available active sites, followed by a slower stage controlled by site saturation and diffusion limitations. The kinetic parameters summarized in Table 2 indicate that the PSO model yields R^2^ closer to unity than the PFO model, demonstrating that the PSO model provides a more accurate description of the adsorption process. This result implies that surface-controlled interactions, potentially involving chemisorption mechanisms such as coordination interactions and π–π stacking, play a dominant role. According to the PSO fitting results, the calculated $q_{e}$ is consistent with the experimental data, further validating the model applicability. The $k_{2}$ reflects the adsorption rate, where a higher $k_{2}$ corresponds to a faster approach to equilibrium.

### 3.4. Comparison of Performance with Other Materials

To assess the adsorption performance of CSAC@MIL-100(Fe), the $q_{m}$ was calculated using the Langmuir isotherm model and compared with previously reported adsorbents for PS removal. The relevant comparative parameters are summarized in Table 4. The $q_{m}$ obtained for CSAC@MIL-100(Fe) is superior to most reported materials under comparable experimental conditions. Beyond its high $q_{e}$, the composite offers additional practical advantages. The synthesis process is straightforward and cost-effective. Moreover, the material exhibits excellent recyclability with minimal performance decay under repeated use. Taken together, these characteristics underscore the strong potential of CSAC@MIL-100(Fe) for practical application in the treatment of PS-contaminated wastewater.

## 4. Conclusions

MPs, owing to their small size, persistence, and resistance to degradation, pose significant environmental and ecological risks. Conventional adsorbents not specifically designed for MP removal often exhibit limited efficiency. In this study, we developed a biomass-derived composite material, CSAC@MIL-100(Fe), via in situ solvothermal growth of MIL-100(Fe) on CSAC, and applied it to investigate polystyrene (PS) microplastics, the most abundant microplastic type in the coastal waters of Hainan. A biomass-derived C framework provided structural stability and macroscopic recoverability, preventing MOF particle aggregation and facilitating physical interception of PS MPs. Meanwhile, MIL-100(Fe) introduced coordination-active sites and aromatic ligands, enabling multiple adsorption interactions, including π–π stacking, electrostatic attraction, and surface complexation. The composite exhibited a high specific surface area of 1050.44 m^2^/g, resulting from the synergistic integration of the porous C matrix and MOF phase. In DI, CSAC@MIL-100(Fe) achieved a removal efficiency of 97.4% and retained 91.44% efficiency after seven cycles, demonstrating excellent recyclability. The $q_{m}$ calculated from the Langmuir model reached 746.27 mg/g. In the competitive adsorption experiments conducted in a binary system containing PS MPs and MB, the presence of MB exerted only a limited inhibitory effect on PS removal under the investigated conditions. In addition, the composite maintained a removal efficiency exceeding 80% across a broad pH range, demonstrating its environmental adaptability and operational stability. The adsorption mechanism was systematically elucidated through comprehensive characterization techniques, including SEM, BET, XRD, FTIR, and XPS analyses, in conjunction with kinetic and isotherm modeling. The equilibrium data were better described by the Freundlich isotherm model, indicating heterogeneous multilayer adsorption, while the kinetic results followed the pseudo-second-order model, suggesting that surface-controlled interactions dominate the adsorption process. Mechanistically, PS immobilization on CSAC@MIL-100(Fe) is governed by synergistic interactions, including π–π stacking between aromatic structures, electrostatic attraction, and coordination interactions involving the metal centers. Overall, the combined advantages of high adsorption capacity, structural stability, recyclability, and tolerance to variable environmental conditions highlight CSAC@MIL-100(Fe) as a promising, cost-effective candidate for practical MP remediation in aquatic systems.

## Figures and Tables

**Table 1:** Pore structure parameters of CSAC, CSAC@MIL-100(Fe)_0.4_, CSAC@MIL-100(Fe)_0.5_, CSAC@MIL-100(Fe)_0.6_, and CSAC@MIL-100(Fe)_0.8_.

Samples	S_BET_ (m^2^/g)	Pore Volume (cm^3^/g)	Average Pore Size (nm)	Type
CSAC	1034.22	0.4224	1.62	I
CSAC@MIL-100(Fe)_0.4_	699.86	0.2858	1.62	I
CSAC@MIL-100(Fe)_0.5_	912.40	0.3766	1.63	I
CSAC@MIL-100(Fe)_0.6_	1050.44	0.4272	1.61	I
CSAC@MIL-100(Fe)_0.8_	1127.68	0.4662	1.64	I

**Table 2:** Isotherm model parameters for PS adsorption on CSAC@MIL-100(Fe) at 25 °C derived from Langmuir and Freundlich fittings.

Sample	Langmuir Model			Freundlich Model		
CSAC@MIL-100(Fe)	$k_{L}$ (L/mg)	$q_{m}$ (mg/g)	R^2^	$k_{F}$ (mg^1−1/n^L^1/n^)/g	n	R^2^
	0.320	746.27	0.938	246.34	5.04	0.999

**Table 3:** Kinetic model parameters for PS adsorption on CSAC@MIL-100(Fe).

Sample	PFO Model				PSO Model		
CSAC@MIL-100(Fe)	C_0_ (mg/mL)	$q_{e}$ (mg/g)	$k_{1}$	R^2^	$q_{e}$ (mg/g)	$k_{2}$	R^2^
	0.1	309.87	0.012	0.987	340.95	0.000051	0.998
	0.3	523.49	0.013	0.986	574.76	0.000032	0.996
	0.5	689.51	0.028	0.984	739.14	0.000051	0.995
	0.7	820.60	0.048	0.984	858.63	0.000083	0.994
	0.9	938.04	0.057	0.982	976.09	0.000090	0.992

**Table 4:** Comparison of $q_{m}$ for PS MPs between CSAC@MIL-100(Fe) and previously reported adsorbents.

Sample	MPs	$q_{m}$ (mg/g)	Removal Rate (%)	References
CSAC@MIL-100(Fe)_0.6_	PS	340.95	97.4	This study
Zn-Al LDH	NPDs	164.49	100	[48]
G@LDO	PS	209.39	80	[49]
MBC	PS	100.60	94.81	[50]
pGel@IPN	PVC	321.81	95	[51]
LPA	PS	368.066	93.68	[52]
LG-GH-PVA@2	PS	288.6	92.7	[53]
Fe_3_O_4_@CMC-MIL-101-NH_2_	PS	245.1	98.0	[54]
PDA-MCS	PE	125	92.3	[55]
MSNC-Fe3	PS	148.39	96.5	[56]
M-MgAl-LDO	PS	203.12	97	[57]

## Data Availability

The original contributions presented in this study are included in the article/Supplementary Material. Further inquiries can be directed to the corresponding authors.

## Supplementary Materials

The following supporting information can be downloaded at https://www.mdpi.com/article/10.3390/polym18060772/s1: Figure S1: Calibration curve of absorbance versus concentration; Figure S2: Zeta potential measurements were performed on the PS spheres to assess their surface charge. The supporting information for this study will be available online upon the publication of the article.
