# Supplementary material for: In Situ Growth of MIL-100(Fe) on Coconut Shell Activated Carbon for High-Efficiently Removal of Microplastics from Water
Source: Polymers (Basel). 2026 Mar 23;18(6):772. doi: 10.3390/polym18060772 (PMC13030399; doi:10.3390/polym18060772)
Supplement: Supplementary file 1 [file polymers-18-00772-s001.zip › polymers-4194601-supplementary.pdf]

Supporting Information

MIL-100(Fe) Supported by Coconut Shell Activated Carbon: Preparation and Adsorption Performance

1. Experimental Section/Methods

1.1 Calibration curve of absorbance versus concentration.

To quantify PS concentration in solution and enable precise analysis of its adsorption, the absorbance of PS suspensions across a concentration gradient was measured using UV-Vis spectrophotometry. This data was used to establish a standard calibration curve relating absorbance to concentration.

1.2 Zeta potential measurement of PS beads.

To investigate the potential electrostatic attraction between PS microspheres and CSAC@MIL-100(Fe)$_{0.6}$, their surface charges were determined via zeta potential measurements.

2. Results

2.1 Calibration curve of absorbance versus concentration.

The absorbance of polystyrene (PS) sphere suspensions at concentrations ranging from 15 to 100 mg/L (specifically, 15, 20, 25, 30, 35, 40, 45, 50, 60, 70, 80, 90, and 100 mg/L) was measured at 260 nm using a UV-Vis spectrophotometer.

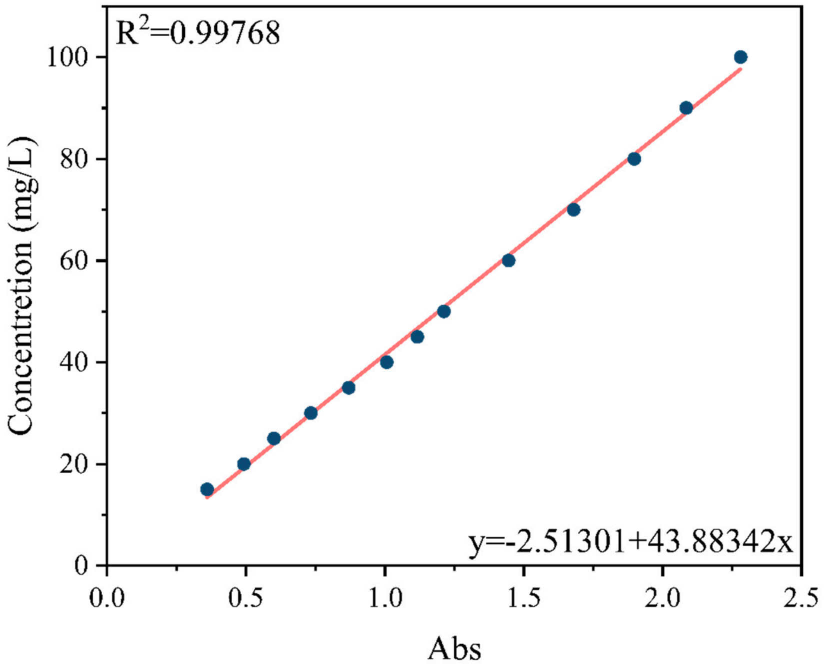

Figure S1. Calibration curve of absorbance versus concentration.

The experimental data revealed a positive correlation between absorbance and concentration, consistent with our hypothesis. A calibration curve was fitted to the data (y = -2.51301 + 43.88342x), thereby establishing a quantitative basis for the subsequent adsorption experiments.

2.2 Zeta potential measurement of PS beads.

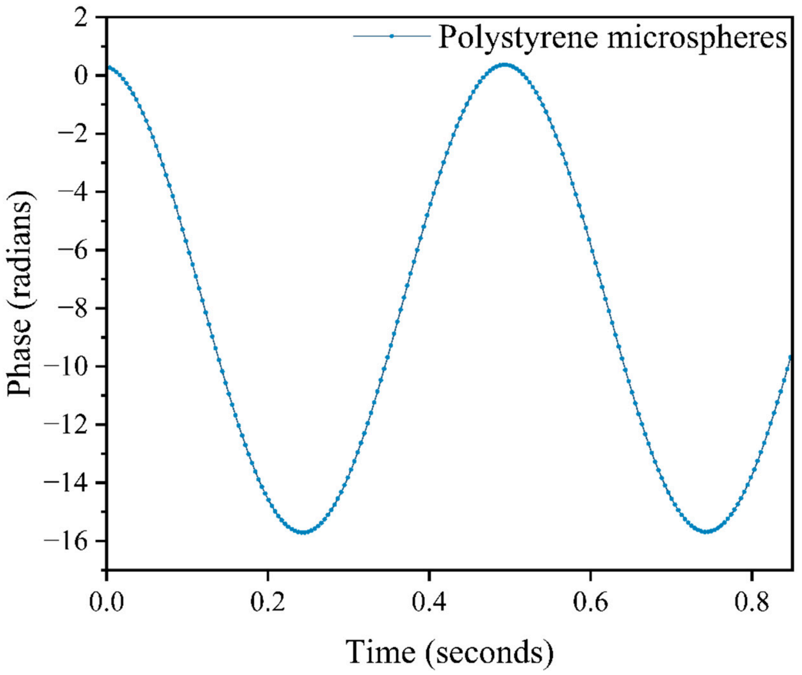

Figure S2. Zeta potential measurements were performed on the PS spheres to assess their surface charge.

The surface charge of the polystyrene (PS) spheres was characterized to elucidate the electrostatic attraction between PS and CSAC@MIL-100(Fe)$_{0.6}$. Specifically, the stock PS suspension was diluted, and its zeta potential was measured in triplicate at pH 7.0 and 25 °C using a Zeta potential analyzer. The measured values were -35.9 mV, -38.46 mV, and -34.92 mV, confirming a consistently negative surface charge.
